# Conjunctival venous malformations: a case report

**DOI:** 10.11604/pamj.2015.22.129.6884

**Published:** 2015-10-13

**Authors:** Elorch Hamza, Alami Fadoua

**Affiliations:** 1 Université Mohammed V Souissi, Service d'Ophtalmologie B, Hôpital des Spécialités CHU, Rabat, Maroc

**Keywords:** Conjunctival venous, malformations, vascular lesion

## Image in medicine

A 37-year-old female presented to the eye emergency department with a conjunctival vascular lesion in here right eye present since birth, remained asymptomatic until 2 months prior to presentation, she then complained of tearing, throbbing pain, and blurred vision following an apparent increase in size. She has no significant past medical or ocular history. Best-corrected visual acuity was 10/10, and intraocular pressure was 12 mm Hg in both eyes. Slit-lamp examination of the right eye revealed a large mobile, lobulated, non pulsatile, red vascular lesion involving a large part of the temporal and nasal quadrants of the globe (A, B, C). The mass extended to the superolateral fornix and was associated with a larg flat subconjunctival hemorrhage (D). The cornea was clear, the iris and the lens were normal. There was no proptosis, globe displacement, or motility disturbance. This is characteristic of periocular vascular malformation and it satisfies the criteria for a venous malformation because of its congenital nature and relative stability. Venous malformations are the rarest of vascular anomalies, especially when situated in the conjunctiva; they are symptomatically distressing and surgically challenging. The present case is congenital; it had been painless and enlarged minimally as she grew, with a more rapid increase in size only in the month before consultation. Based on this fact, we decided for a surgical excision of the lesion with secondary reconstruction.

**Figure 1:**
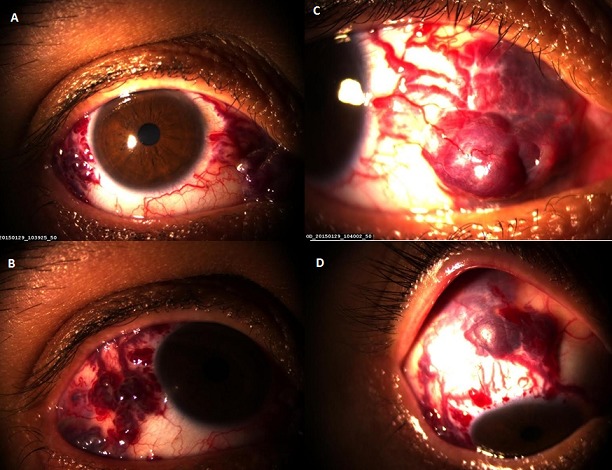
(A) conjunctival venous malformation: a reddish-blue multilobular, movable mass in the temporal and nasal quadrant of the right eye; (B) close-up view of the lesion in the temporal quadrant of the right eye with irregular epibulbar margin; (C) close-up view of the lesion in the nasal quadrant of the right eye; (D) mass extended to the superolateral fornix and was associated with a larg flat subconjunctival hemorrhage

